# Complete Genome Sequence of Tomato Mosaic Virus Isolated from Jasmine in the United States

**DOI:** 10.1128/genomeA.00706-15

**Published:** 2015-07-09

**Authors:** Kornelia Fillmer, Scott Adkins, Patchara Pongam, Tom D’Elia

**Affiliations:** a Department of Biology, Indian River State College, Fort Pierce, Florida, USA; b United States Department of Agriculture, Agricultural Research Service, Horticultural Research Laboratory, Fort Pierce, Florida, USA

## Abstract

Tomato mosaic virus was reported from jasmine in Florida. We present the first complete genome sequence of a tomato mosaic virus isolate from this woody perennial plant in the United States.

## GENOME ANNOUNCEMENT

The genus *Tobamovirus* in the family *Virgaviridae* is composed of viruses with rigid rod-shaped virions that are easily transmitted by mechanical inoculation and plant-to-plant contact (1). Currently, there are 35 species in the genus *Tobamovirus* (http://www.ictvonline.org/virusTaxonomy.asp), which are distributed worldwide and include tobacco mosaic virus, tomato mottle mosaic virus, and hibiscus latent Fort Pierce virus. These tobamoviruses commonly infect herbaceous annual plant species, including tomato and pepper, and woody perennial plant species, including hibiscus (2–4). Tomato mosaic virus (ToMV), another tobamovirus species, has consistently been identified as infecting the ornamental plant jasmine (*Jasminum multiflorum*) in Florida since 1999 (5). Here, we present the first complete genome sequence of a ToMV isolate from jasmine in Florida.

Tissue (leaf, bark, and flower) from symptomatic jasmine was used initially to mechanically inoculate *Nicotiana debneyi*. Symptomatic *N. debneyi* tissue was used to subsequently mechanically inoculate *N. tabacum* var. Xanthi, from which ToMV virions were partially purified from systemically infected tissue using a typical tobamovirus protocol (6). ToMV genomic RNA was extracted from virions using TRIzol reagents (Invitrogen, Carlsbad, CA, USA). A cDNA library was prepared using Ion Total RNA sequencing (RNA-Seq) version 2 and Ion Xpress RNA-Seq bar-code kits (Life Technologies, Carlsbad, CA, USA) and subsequently sequenced using an Ion PGM Template OT2 200 kit with an Ion 314 Chip on an Ion Torrent Personal Genome Machine (Life Technologies), according to the manufacturer's protocols. Individual reads were mapped using the International Committee on Taxonomy of Viruses reference sequence for ToMV (GenBank accession no. AF332868) as a scaffold with the Torrent Mapping Alignment Program (TMAP) version 4.21 for Ion Torrent (Thermo Fisher Scientific, Waltham, MA). Geneious R8 version 8.1.4 (Biomatters, Auckland, New Zealand) and Integrative Genomics Viewer (IGV) version 2.3 (7, 8) were used to obtain a complete consensus sequence.

Comparative analysis showed that the Florida ToMV isolate sequence (6,383 nucleotides) was 99% identical to the reference ToMV sequence with 69 single-nucleotide differences. Of the 49 nucleotide differences within the predicted four tobamovirus open reading frames (126- and 183-kDa replication proteins, movement protein, and coat protein), 46 were silent, whereas 3 led to amino acid changes.

### Nucleotide sequence accession number. 

The complete genome sequence of the jasmine ToMV isolate was deposited in GenBank under the accession no. KR537870.
